# Nitrogen Loss from Pristine Carbonate-Rock Aquifers of the Hainich Critical Zone Exploratory (Germany) Is Primarily Driven by Chemolithoautotrophic Anammox Processes

**DOI:** 10.3389/fmicb.2017.01951

**Published:** 2017-10-10

**Authors:** Swatantar Kumar, Martina Herrmann, Bo Thamdrup, Valérie F. Schwab, Patricia Geesink, Susan E. Trumbore, Kai-Uwe Totsche, Kirsten Küsel

**Affiliations:** ^1^Aquatic Geomicrobiology Group, Institute of Biodiversity, Friedrich Schiller University Jena, Jena, Germany; ^2^Department of Biogeochemical Processes, Max-Planck-Institute for Biogeochemistry, Jena, Germany; ^3^German Center for Integrative Biodiversity Research (iDiv) Halle-Jena-Leipzig, Leipzig, Germany; ^4^Department of Biology, Nordic Center for Earth Evolution, University of Southern Denmark, Odense, Denmark; ^5^Institute of Inorganic and Analytical Chemistry, Friedrich Schiller University Jena, Jena, Germany; ^6^Hydrogeology, Institute of Geosciences, Friedrich Schiller University Jena, Jena, Germany

**Keywords:** anammox, chemolithoautotrophy, denitrification, groundwater, ladderane lipids, subsurface

## Abstract

Despite the high relevance of anaerobic ammonium oxidation (anammox) for nitrogen loss from marine systems, its relative importance compared to denitrification has less been studied in freshwater ecosystems, and our knowledge is especially scarce for groundwater. Surprisingly, phospholipid fatty acids (PLFA)-based studies identified zones with potentially active anammox bacteria within two superimposed pristine limestone aquifer assemblages of the Hainich Critical Zone Exploratory (CZE; Germany). We found anammox to contribute an estimated 83% to total nitrogen loss in suboxic groundwaters of these aquifer assemblages at rates of 3.5–4.7 nmol L^−1^ d^−1^, presumably favored over denitrification by low organic carbon availability. Transcript abundances of *hzsA* genes encoding hydrazine synthase exceeded *nirS* and *nirK* transcript abundances encoding denitrifier nitrite reductase by up to two orders of magnitude, providing further support of a predominance of anammox. Anammox bacteria, dominated by groups closely related to *Cand*. Brocadia fulgida, constituted up to 10.6% of the groundwater microbial community and were ubiquitously present across the two aquifer assemblages with indication of active anammox bacteria even in the presence of 103 μmol L^−1^ oxygen. Co-occurrence of *hzsA* and *amoA* gene transcripts encoding ammonia mono-oxygenase suggested coupling between aerobic and anaerobic ammonium oxidation under suboxic conditions. These results clearly demonstrate the relevance of anammox as a key process driving nitrogen loss from oligotrophic groundwater environments, which might further be enhanced through coupling with incomplete nitrification.

## Introduction

Over the last decades, human impact on the nitrogen cycle has resulted in increasing concentrations of nitrate in groundwater, which is of growing concern on a global scale (Galloway, 2005; Burgin and Hamilton, 2007; Schlesinger, 2009). However, sources and sinks of nitrate in aquifers and the interconnecting biogeochemical processes are still not fully understood. Globally, 25% of the drinking water for the human population originates from karstic aquifers, which are especially vulnerable to nitrate contamination due to their potential for rapid infiltration and temporary inflow of oxygenated water (Auckenthaler et al., 2002; Ford and Williams, 2007; Huebsch et al., 2014).

Traditionally, nitrogen losses from freshwater environments including aquifers have primarily been attributed to heterotrophic denitrification (Seitzinger et al., 2006; Burgin and Hamilton, 2007; Rivett et al., 2008). However, especially under conditions of organic carbon limitation as they may occur in pristine limestone aquifers, autotrophic nitrate reducing processes such as autotrophic denitrification or anaerobic oxidation of ammonium (anammox) are likely to become more competitive. Burgin and Hamilton (2007) suggested that the relative availability of labile carbon or reduced sulfur and iron as potential inorganic electron donors for chemolithoautotrophic denitrification are the key determinants of nitrate removal pathways. Despite the high relevance of anammox for nitrogen losses from marine systems, e.g., oxygen minimum zones (Thamdrup and Dalsgaard, 2002; Jensen et al., 2011; Lam and Kuypers, 2011), this process has only recently become the focus of studies addressing nitrogen loss from freshwater or semiterrestrial environments (Schubert et al., 2006; Clark et al., 2008; Moore et al., 2011; Yoshinaga et al., 2011; Yang et al., 2015; Zhu et al., 2015; Shen et al., 2016). Anammox bacteria thrive in low temperature environments, which makes groundwater a suitable environment for anammox to occur (Dalsgaard and Thamdrup, 2002; Rysgaard and Glud, 2004; Isaka et al., 2008; Canion et al., 2014). In fact, isotope-based studies and molecular surveys provided first evidence of the potential for anammox in groundwater environments (Clark et al., 2008; Smits et al., 2009; Humbert et al., 2010). Subsequent studies suggested that anammox could be an important process responsible for nitrogen loss from ammonium- and nitrate-contaminated groundwater with up to 90% of nitrogen loss being attributed to anammox (Clark et al., 2008; Moore et al., 2011; Robertson et al., 2012; Hanson and Madsen, 2015; Smith et al., 2015).

Two recent studies carried out within the carbonate-rock aquifer assemblages of the Hainich Critical Zone Exploratory (Thuringia, Germany; Küsel et al., 2016) provided first evidence of active anammox bacteria in uncontaminated, oligotrophic groundwater (Schwab et al., 2017; Starke et al., 2017). These two superimposed aquifer assemblages are largely pristine with low microbial biomass, very low concentrations of organic carbon, and limited impact of agricultural land-use on groundwater nitrate concentrations (Kohlhepp et al., 2016; Küsel et al., 2016). Based on the presence of [3]-ladderane and [5]-ladderane phospholipid derived fatty acids (PLFAs), Schwab et al. (2017) suggested an important role of anammox in anoxic groundwater of these assemblages at higher ${\text{NH}}_{4}^{+}$ concentrations, which was further supported by a metaproteomics study showing that one third of the identified protein groups in anoxic groundwater samples was associated with Brocadiales (Starke et al., 2017). However, the role of anammox compared to denitrification for nitrogen loss from this oligotrophic aquifer system has remained unclear. In this study, we aimed to assess the relevance of anammox vs. denitrification by rate measurements at a representative site for which these previous studies suggested a high potential for anammox, and determine the genetic potential for anammox and denitrification across the two aquifer assemblages. While studies of marine environments suggested that nitrite originating from incomplete nitrification may fuel the anammox process in oxygen minimum zones (Lam et al., 2007, 2009), the relevance of a potential coupling of these two processes for the removal of fixed nitrogen from groundwater environments has not yet been addressed. Consequently, we also aimed to analyze potential links between anammox and nitrification targeting transcriptional activity of genes involved in anammox and aerobic ammonia oxidation.

## Methods

### Study site, sample collection, and chemical analysis

**Figure 1:**
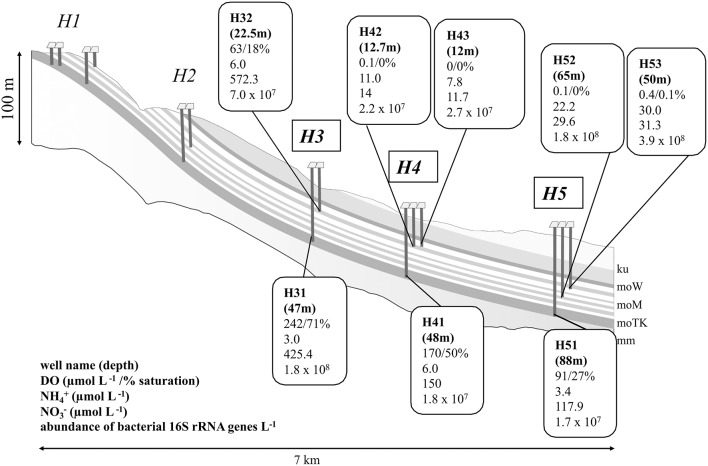
Characteristics of the Hainich aquifer assemblages. Sampling sites investigated in this study (encircled) included five wells from the Hainich transect upper aquifer assemblage (HTU): H32, H42, H43, H52, H53, and three wells from the Hainich transect lower aquifer assemblage (HTL): H31, H41, H51. Parameters and units presented per well are explained at the bottom in the left corner. Data represent means of samples obtained between January 2014 and June 2015 (*n* = 19). More information about groundwater hydrochemistry is provided in Supplementary Table 1. ku, Lower Keuper; moW, Warburg formation; moM, Meissner formation; moTK, Trochitenkalk formation; mm, Middle Muschelkalk; based on Kohlhepp et al. (2016).

Groundwater samples were obtained from groundwater ecosystems in the temperate carbonate-rock terrain of the Hainich Critical Zone Exploratory (CZE) located in Thuringia, Germany. A monitoring well transect offers access to two superimposed limestone aquifer assemblages, established in the framework of the Collaborative Research Center (CRC) AquaDiva (Küsel et al., 2016). The location, geological setting, and groundwater well construction have been described in more detail by Küsel et al. (2016). Bedrocks containing the aquifers of the investigated area belong to the lithostratigraphic subgroup Upper Muschelkalk of the German Triassic (Kohlhepp et al., 2016). Here, superimposed aquifer assemblages are developed in alternating sequences of fractured limestones (fracture aquifers) and marlstones (aquitards), aggregated to the upper aquifer assemblage (HTU: wells H32, H42, H43, H52, H53) and the limestone-dominated lower aquifer assemblage (HTL: wells H31, H41, H51; Küsel et al., 2016; Figure 1) with recharge areas covered by forest, pastures, or cropland (HTU) or mostly forest (HTL) (Kohlhepp et al., 2016; Küsel et al., 2016).

Within the coordinated long-term monitoring program of the CRC AquaDiva, regular sampling of groundwater is carried out, which allowed access to monthly groundwater samples from January 2014 to August 2015 and additionally from November 2015 for this study. Samples were obtained from eight wells using submersible motor pumps (MP1, Grundfos, Denmark) after steady state in the physical and chemical conditions were established. Water temperature, dissolved oxygen concentration, pH, and redox potential were measured in a flow-through cell in the field using respective probes (Küsel et al., 2016). Groundwater was then filtered through 0.2 μm-pore size sterile polyvinylidine fluoride (PVDF) syringe filters for subsequent analyses of nitrate, nitrite and ammonium concentrations using standard colorimetric procedures (DEV, 1975; Grasshoff et al., 1983). The concentration of sulfide was determined by the modified methylene blue method after fixation of samples with 2% (v/v) Zn-acetate (Trüper and Schlegel, 1964). Concentrations of sulfate were determined by ion chromatography [IC 20 system (Dionex, Sunnyvale, CA) equipped with an IonPac AS11-HC column and an IonPac AG11-HC precolumn], and total organic carbon (TOC) concentrations were determined by using a TOC analyzer (AnalytikJena, Germany). Concentrations of Ca and K were determined by ICP-OES (725 ES, Varian/Agilent, USA) after filtration through 0.45 μm pore size polyether-sulfone (PES) filters (Kohlhepp et al., 2016). Groundwater samples for molecular analysis were transferred to sterile glass bottles, filled up to the maximum filling level, and transported to the laboratory at 4°C, followed by filtration through 0.2 μm PES filters (Supor, Pall Corporation, USA) for DNA extraction and through 0.2 μm polycarbonate filters (Nuclepore, Whatman; Merck-Millipore) for RNA extraction within 1 h, with 5–6 L of groundwater passing through one filter. The filters were then transferred to sterile 2 ml tubes and frozen on dry ice within 1 min, followed by storage at −80°C. For sampling of groundwater particulate organic matter (POM) for later PLFA analysis, ~1,000 L of groundwater were filtered on site using a stainless steel filter holder (diameter 293 mm; Millipore, USA) equipped with a removable pre-combusted (5 h at 500°C) glass fiber membrane filter (pore size 0.3 μm; Sterlitech, USA; Schwab et al., 2017).

### Ladderane lipids extractions and measurements

Ladderane phospholipid derived fatty acids (PLFA) were extracted from glass fiber filters and subsequently purified using a slightly modified method of the common PLFA extraction method, as previously described (Schwab et al., 2017). The ladderane FAMEs were identified based on published mass spectra (Sinninghe Damsté et al., 2005) using a gas chromatograph (Trace 1310 GC) coupled to a triple quadrupole mass spectrometer (TSQ-8000; Thermo-Fisher, Bremen, Germany). While Schwab et al. (2017) expressed ladderanes in percentage relative to the other measured PLFAs, we used a different calculation procedure to allow a better estimation of differences in the relative abundance of active anammox bacteria between the different wells. Here, the peak area of the most abundant ladderanes, C_20_[5]-ladderane and C_20_[3]-ladderane, relative to the peak area of the internal standard C19:0 FAME was used as an alternative calculation. Ladderane concentrations were calculated relative to the internal standard nonadecanoic acid-methyl ester (19:0) added prior to GC analysis and relative to a standard mixture (FAME-Mix, Thermo-Fisher, Bremen, Germany) measured in 5 different concentrations between 2 and 40 ng/μl. This approach required a recalculation of ladderane data already presented in Schwab et al. (2017) (samples from July, September, and December 2014), and additional data from a new time point (November 2015) were integrated in this study.

### Anammox and denitrification rate measurements

A ^15^N-labeling approach (Dalsgaard et al., 2012) was used to measure anammox and denitrification rates in the groundwater samples from well H53 in November 2015, for which a previous PLFA-based study had suggested high abundances of anammox bacteria (Schwab et al., 2017). Anoxic incubations were set up using 30 ml of groundwater per incubation in serum bottles. During sampling, the groundwater sample was allowed to flow through a sterile pipette to the bottom of a 1 L sterile glass bottle, followed by overflow for three volume exchanges, leaving the bottles without headspace. The bottles were then closed with rubber stoppers and transferred to the laboratory at 4°C within 2 h. All the water samples were flushed with nitrogen, followed by immediate processing inside an anaerobic chamber, where each 30 ml of the water samples were dispensed into serum bottles with 8 ml headspace volume. ^15^N-labeled ${\text{NH}}_{4}^{+}$ and ${\text{NO}}_{2}^{-}$ were added as two treatments: (1) ^15^${\text{NH}}_{4}^{+}$ (50 μM) and ^14^${\text{NO}}_{2}^{-}$ (5 μM); (2) ^15^${\text{NO}}_{2}^{-}$ (5 μM) without adding additional ^14^${\text{NH}}_{4}^{+}$. The headspace of the serum bottles was purged with helium gas for 5 min. The bottles were incubated at 15°C in the dark, and triplicate serum bottles were destructively sampled after 0, 14, 24, 36, and 48 h by introducing 300 μl of saturated aqueous Zinc chloride solution. The isotopic composition of the N_2_ gas in the headspace of each sample was measured by coupled gas chromatography isotope ratio mass spectrometry (Dalsgaard et al., 2012). Rates of anammox and denitrification were derived from the accumulation of the ^15^N-labeled N_2_ species ^14^N^15^N (^29^N_2_) and ^15^N^15^N (^30^N_2_) as previously described (Thamdrup and Dalsgaard, 2002). For the calculation of rates of anammox and denitrification, the dilution of the ^15^N label by unlabeled (background) nitrite and ammonium already present was taken into account, with the labeled fraction determined as the ratio of the concentration of ^15^N labeled nitrite or ammonium added to the total concentration of nitrite or ammonium, respectively. For nitrite, the total concentration was determined as the sum of the background concentration and the concentration of ^15^N added, and for ammonium, the total concentration was measured after addition of ^15^${\text{NH}}_{4}^{+}$.

### Nucleic acid extraction, PCR amplification, and cloning

Genomic DNA and total RNA were extracted using the PowerSoil DNA isolation kit (MO BIO Laboratories Inc., USA) and the PowerWater RNA Isolation Kit (MO BIO Laboratories Inc., USA), respectively, according to the manufacturer's protocol. Processing of RNA was performed as described previously (Schwab et al., 2017). PCR for later clone library construction to generate standards for quantitative PCR was carried out using HotstarTaq Mastermix (Qiagen, Germany) with previously published primer combinations and cycling conditions: 526F/1857R5 for *hzsA* genes encoding hydrazine synthase subunit A of anammox bacteria (Harhangi et al., 2012), and F1aCu/R3Cu and cd3aF/R3cd, respectively, for *nirK* and *nirS* genes encoding copper- and cytochrome c-dependent nitrate reductase of denitrifiers (Hallin and Lindgren, 1999; Michotey et al., 2000; Throbäck et al., 2004), following the cycling conditions given in Throbäck et al. (2004). Clone libraries were constructed using pGEM T-Easy cloning vector and chemically competent *Escherichia coli* (JM109) in accordance with the manufacturer's protocols (Promega). Plasmids were extracted using GeneJET plasmid miniprep kit (Thermo Fisher Scientific, Germany) and sequencing of cloned inserts was performed at Macrogen (The Netherlands).

### Quantitative PCR

Quantification of bacterial 16S rRNA genes, and *hzsA, nirK, nirS*, and *amoA* genes and transcripts was performed by quantitative PCR (qPCR) on a Mx3000P qPCR cycler (Agilent Technologies) using Maxima SYBR Green Mastermix (Thermo Fisher Scientific). QPCR targeting bacterial 16S rRNA genes, *hzsA, nirK*, and *nirS* genes was performed for monthly samples from January 2014 to August 2015, while qPCR targeting *amoA* genes and transcripts of *hzsA, nirK, nirS*, and *amoA* was only performed for samples obtained in August and November 2015. In detail, the following primer combinations were used: Bac8Fmod/Bac338Rabc (Daims et al., 1999; Loy et al., 2002) for bacterial 16S rRNA genes following Herrmann et al. (2012), 1597F/1857R5 for *hzsA* genes (Harhangi et al., 2012), F1aCu/R3Cu and cd3aF/R3cd, respectively, for *nirK* and *nirS* genes (Hallin and Lindgren, 1999; Michotey et al., 2000; Throbäck et al., 2004) with cycling conditions as given in Throbäck et al. (2004), and primers Arch-AmoAF/Arch-AmoAR (Francis et al., 2005) and AmoA-1F/AmoA-2R (Rotthauwe et al., 1997) for archaeal and bacterial *amoA* genes as described in Opitz et al. (2014). Standard curves were produced based on a serial dilution of non-linearized plasmids containing inserts of the respective target genes and were linear from 5 × 10^1^ to 5 × 10^8^ copies per reaction with *R*^2^ > 0.99) for the functional genes and from 5 × 10^2^ to 5 × 10^8^ copies per reaction for bacterial 16S rRNA genes.

### Illumina MiSeq amplicon sequencing

16S rRNA-gene based analysis to assess the structure and taxonomic affiliation of the total bacterial community was performed for samples taken in November 2015 when anammox rate measurements were carried out. To confirm the observed community patterns, we performed additional 16S rRNA gene-targeted amplicon sequencing from previous time points to link community structure information to the quantification of functional gene transcripts (August 2015) and to characterize the total bacterial population and the population with the potential for protein biosynthesis (Blazewicz et al., 2013) by comparing DNA- and RNA-based 16S rRNA amplicon data from the same sample (August 2014). Bacterial 16S rRNA genes were targeted using the primer combination Bakt_341F/Bakt_805R (Herlemann et al., 2011), covering the V3–V5 region of the bacterial 16S rRNA gene. Amplicons of *nirS* and *nirK* genes were generated from genomic DNA only (August 2014) using the same primer combinations as for cloning and qPCR. Generation of barcoded amplicons and amplicon sequencing using the Illumina MiSeq platform was performed by LGC Genomics (Berlin). The PCRs included about 5 ng of DNA extract, 15 pmol of the respective forward and reverse primer in 20 uL volume of 1 × MyTaq buffer containing 1.5 units MyTaq DNA polymerase (Bioline) and 2 μl of BioStabII PCR Enhancer (Sigma). For each sample, the forward and reverse primers had the same 10-nt barcode sequence. PCRs were carried out for 30 cycles using the following parameters: 2 min 96°C predenaturation; 96°C for 15 s, 50°C for 30 s, 70°C for 90 s. For *nirS*, cycling conditions were similar but annealing was at 56°C. About 20 ng amplicon DNA of each sample were pooled for up to 48 samples carrying different barcodes. The amplicon pools were purified with one volume AMPure XP beads (Agencourt) to remove primer dimer and other small mispriming products, followed by an additional purification on MinElute columns (Qiagen). About 100 ng of each purified amplicon pool DNA was used to construct Illumina libraries using the Ovation Rapid DR Multiplex System 1–96 (NuGEN). Illumina libraries were pooled and size selected by preparative Gelelectrophoresis. Sequencing was done on an Illumina MiSeq using V3 Chemistry (Illumina).

### Sequence analysis

Sequence analysis of bacterial 16S rRNA amplicons was performed using Mothur (Schloss et al., 2009, v.1.39.1), following the Mothur MiSeq SOP (Kozich et al., 2013) along with the SILVA bacteria reference alignment (Quast et al., 2013). Taxonomic classification was done using the classify.seqs command in Mothur with a reference database based on SILVA release v128. Species-level operational taxonomic units (OTUs) were assigned using a 0.03 distance cut-off. For comparisons of community structure across samples, the library size of each sample was normalized to the same number of reads using the sub.sample command implemented in Mothur.

Because of insufficient amplification of *nirK* genes for MiSeq Illumina sequencing at very low *nirK* gene abundances in the groundwater, a detailed analysis of denitrifier community composition focused on *nirS*-type denitrifiers only. *nirS* sequences were analyzed using Mothur with few modifications necessary to adjust the pipeline for the analysis of protein-encoding genes, integrating BioEdit (Hall, 1999), and the ARB package (Ludwig et al., 2004). After removing low quality sequence reads following the standard settings of the Mothur MiSeq SOP, nucleic acid sequences were translated to deduced amino acid sequences using BioEdit, and sequences containing stop codons were removed. Nucleic acid sequences were then aligned to a *nirS* reference alignment generated in ARB (Herrmann et al., 2017), and badly aligned sequences were excluded from further analysis. OTU assignment was done at a 0.18 distance cut-off on nucleic acid level (Palmer et al., 2012). Closest relatives were determined using nucleotide BLAST (blastn) of one representative sequence per OTU against the nucleotide collection (nr/nt) database at the National Center for Biotechnology Information (NCBI; https://blast.ncbi.nlm.nih.gov/Blast.cgi). For phylogenetic analysis of cloned *hzsA* fragments, reference alignments of deduced hzsA protein sequences were generated in ARB. Sequences obtained in this study were translated to deduced protein sequences and aligned against the reference alignment. Species-level OTUs were assigned based on a 0.03 distance cut-off on protein level. Closest relatives were determined based on a BLAST search using blastx against the non-redundant protein sequence (nr) database at NCBI (https://blast.ncbi.nlm.nih.gov/Blast.cgi) and using phylogenetic tree construction in ARB. Sequences obtained in this study have been submitted to the European Nucleotide Archive (ENA) for results of MiSeq Illumina amplicon sequencing (study accession number: PRJEB20223, sample accession numbers ERS1645471–ERS1645508) and to Genbank for clone library-based sequencing (accession numbers KY887284–KY887452 for *hzsA* genes, KY887453–KY887461 for *nirS* genes, and KY887462–KY887471 for *nirK* genes).

## Results

### Elucidation and characterization of potential anammox sites

Confirming previous observations (Kohlhepp et al., 2016; Küsel et al., 2016), the groundwater is characterized by a slightly alkaline pH (7.1–7.3 across all wells), low concentrations of total organic carbon (TOC: 1.7–2.2 mg L^−1^) and generally low microbial biomass as approximated by ~10^8^ bacterial 16S rRNA genes per L. The two aquifer assemblages differ strongly in oxygen availability with suboxic (<10 μmol oxygen L^−1^) to anoxic conditions in the upper aquifer assemblage (HTU) and oxic conditions in the lower aquifer (HTL) (Figure 1). Similarly, nitrate concentrations are higher in the HTL wells compared to the HTU wells except well H32, which shows the highest nitrate concentration, while ammonium concentrations are higher in HTU compared to the wells of HTL with highest concentrations of 30 μmol L^−1^ in the groundwater of well H53 (Figure 1).

**Figure 2:**
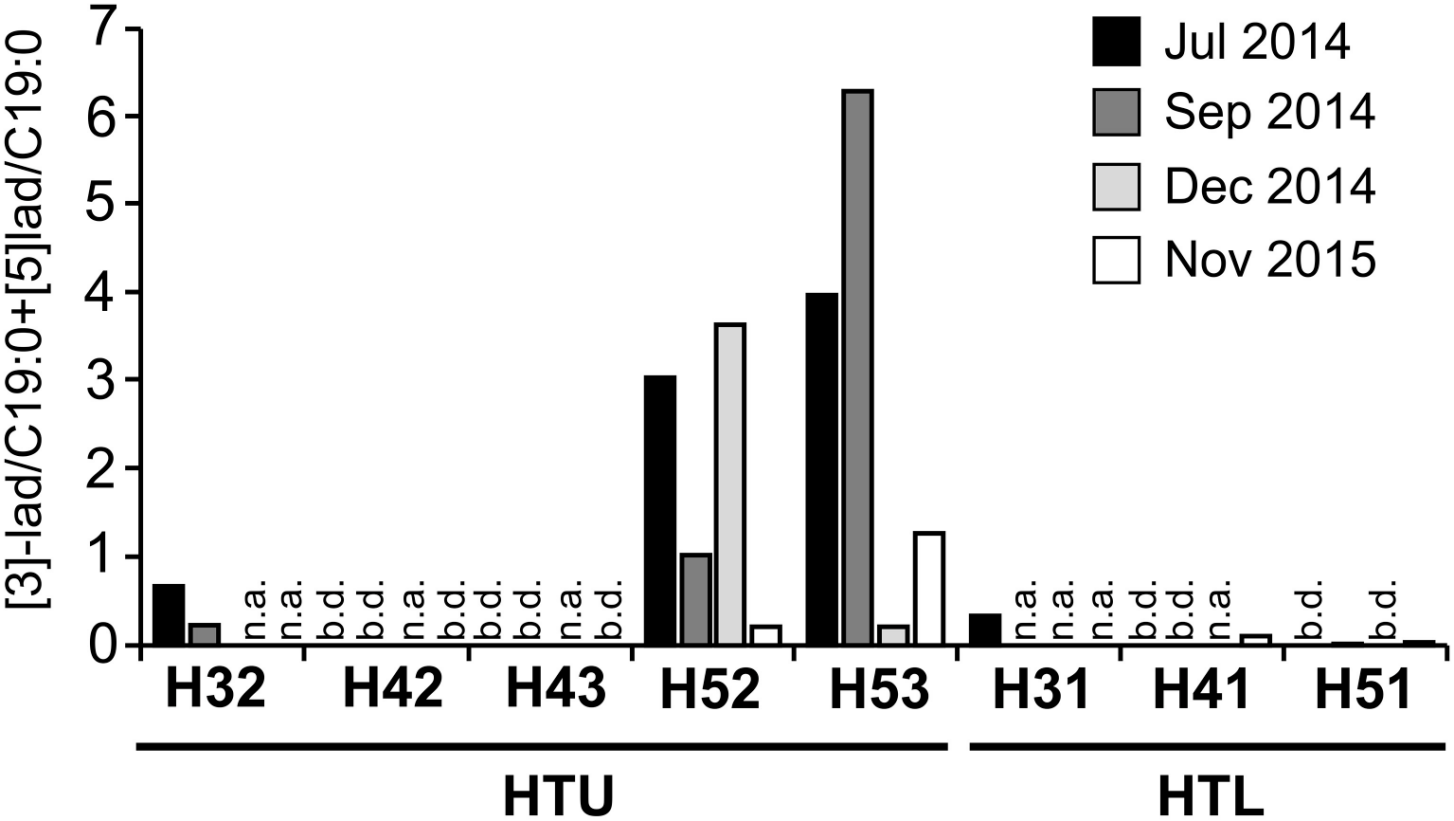
Relative concentrations of ladderane lipids (sum of ladderane-[3]-FAME and ladderane-[5]-FAME peak area relative to C19:0 internal standard peak area) in the groundwater of eight wells along the Hainich groundwater observation transect. b.d., below detection (relative concentrations < 0.01); n.a., not analyzed. Data of July, September, and December 2014 were also subject of analyses published in Schwab et al. (2017).

The highest relative concentrations of ladderanes were observed in the suboxic to anoxic groundwater of wells H52 and H53 with maxima observed at well H53 for most of the time points, as previously shown for samples obtained in July, September, and December 2014 (Schwab et al., 2017). Ladderane data of November 2015 were added here to confirm these patterns for the time point when anammox rate measurements were performed. Interestingly, ladderanes were also detectable in the oxic groundwater of wells H31, H41, and H51, albeit at much lower relative concentrations (Figure 2). Ladderane concentrations found at well H53 exceeded those found in oxic groundwater at H51 by a factor of 35. Since absolute quantification of phospholipids derived [5]- and [3]-ladderanes was not possible due to the absence of commercially available standards, only changes of their relative concentration between wells are presented here. Temporal fluctuations of ladderane concentrations at wells H52 and H53 were more than one order of magnitude but did not show any correlation with temporal fluctuations of oxygen or ammonium concentrations (data not shown).

**Figure 3:**
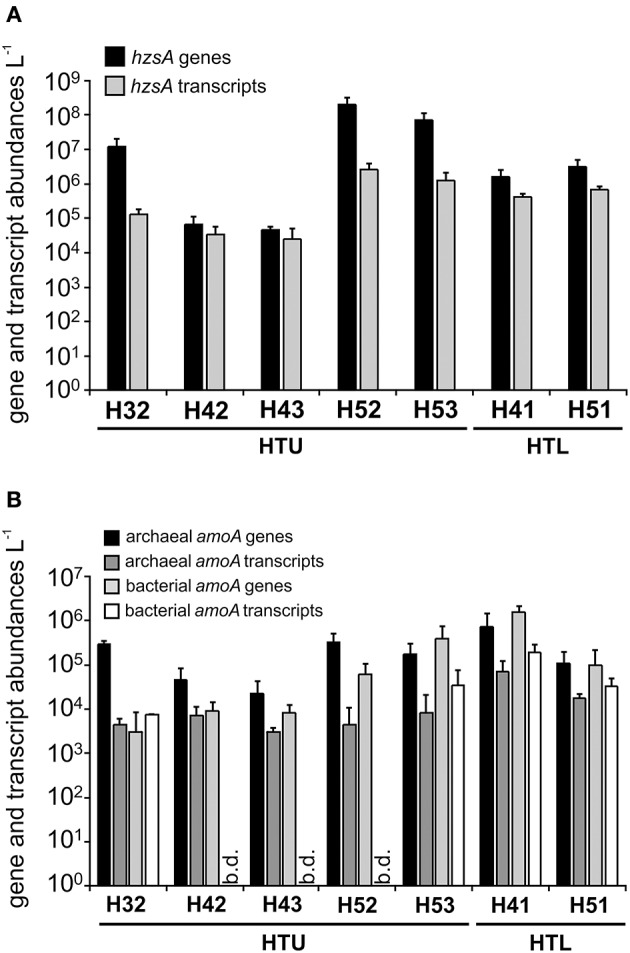
Abundances of genes and transcripts of **(A)** *hzsA* and **(B)** archaeal and bacterial *amoA* in groundwater samples obtained from seven wells of the upper and lower aquifer assemblage in August and November 2015. Bars represent mean (±standard deviation) of two time points and each three technical replicates in qPCR analysis. b.d., below detection.

The ladderane-based results agreed well with the distribution patterns of total and active anammox bacteria suggested by quantitative analysis targeting *hzsA* genes and transcripts, which revealed maximum *hzsA* gene and transcript abundances in the groundwater of wells H52 and H53 of the upper aquifer assemblage (Figure 3A). Based on these observations, we selected well H53 for rate measurements of anammox and denitrification activity. Incubations with ^15^${\text{NH}}_{4}^{+}$ and ^14^${\text{NO}}_{2}^{-}$ or ^15^${\text{NO}}_{2}^{-}$ and natural ${\text{NH}}_{4}^{+}$ background showed a linear increase in ^29^N_2_ concentrations in the headspace (Supplementary Figure 1) and yielded rates of anammox of 4.7 and 3.5 nmol N_2_ L^−1^ d^−1^, respectively, while denitrification was detected at an activity of 0.7 nmol N_2_ L^−1^ d^−1^. Based on the measured activities in the incubations with added ^15^${\text{NO}}_{2}^{-}$, we observed a total N_2_ production activity of 4.2 nmol N_2_ L^−1^ d^−1^ to which anammox contributed an estimated 83%.

### Co-occurrence with denitrifiers and aerobic ammonia oxidizers based on functional genes

Abundances of *nirK* and denitrifier *nirS* genes ranged from 1.1 × 10^3^ to 6.5 × 10^5^ and 1.1 × 10^4^ to 8.5 × 10^7^ genes L^−1^, respectively, across all sites and time points with *nirS* usually outnumbering *nirK* genes by two orders of magnitude, pointing to a strong predominance of *nirS*-type denitrifiers in the groundwater denitrifier communities (Supplementary Figure 2A). Relative proportions of denitrifiers approximated by *nirS*/16S rRNA gene ratios followed a similar trend as observed for the anammox population with maximum gene ratios of 0.068 and 0.095 at suboxic to anoxic wells H52 and H53, respectively, and lower ratios in the anoxic groundwater at site 4 (0.014 and 0.015 at H42 and H43), or in the oxic wells (H31, H32, H41, H51: 0.028–0.046; Supplementary Figure 2B). Transcript abundances of *nirS* and *nirK* remained below the quantification limit of 10^3^ transcripts L^−1^ groundwater for all wells (data not shown).

To get first insight into potentially co-occurring activities of aerobic and anaerobic ammonium oxidation, we quantified *amoA* genes and transcripts of ammonia-oxidizing archaea and bacteria for seven groundwater wells in August and November 2015. *amoA* gene abundances pointed to similar or only slightly lower total abundances of aerobic ammonia oxidizers in suboxic to anoxic wells H52 and H53 compared to the oxic well H41 and to even higher abundances compared to oxic well H51 (Figure 3B). However, comparison of *amoA* gene abundances to bacterial 16S rRNA gene abundances suggested a smaller relative fraction of ammonia oxidizers within the total microbial communities of wells H52 and H53 compared to wells H41 and H51 (data not shown). For both time points, transcripts of bacterial and archaeal *amoA* genes were detectable in the oxic groundwater of HTL but also at H52 and H53. We calculated gene and transcript ratios of [*hzsA*/sum of archaeal and bacterial *amoA*] across sites as an indicator of a predominance of either anaerobic or aerobic ammonium oxidation in the genetic potential or transcriptional activity of the groundwater microbial communities. *hzsA*/*amoA* ratios ranged from 0.1 to 444.7 and from 1.2 to 1087.6 on the gene and transcript level, respectively (Supplementary Figure 3). Highest *hzsA*/*amoA* gene and transcript ratios were observed for wells H52 and H53. *hzsA*/*amoA* ratios were negatively correlated to groundwater oxygen concentrations across sites (Spearman rank correlation coefficient −0.85 and −0.875 for gene and transcript-based analysis, respectively, *p* < 0.01; Supplementary Figure 4) when excluding wells H42 and H43 where *hzsA* and *amoA* transcript numbers were close to the detection limit.

### Identification of key organisms responsible for nitrogen loss in the groundwater

**Figure 4:**
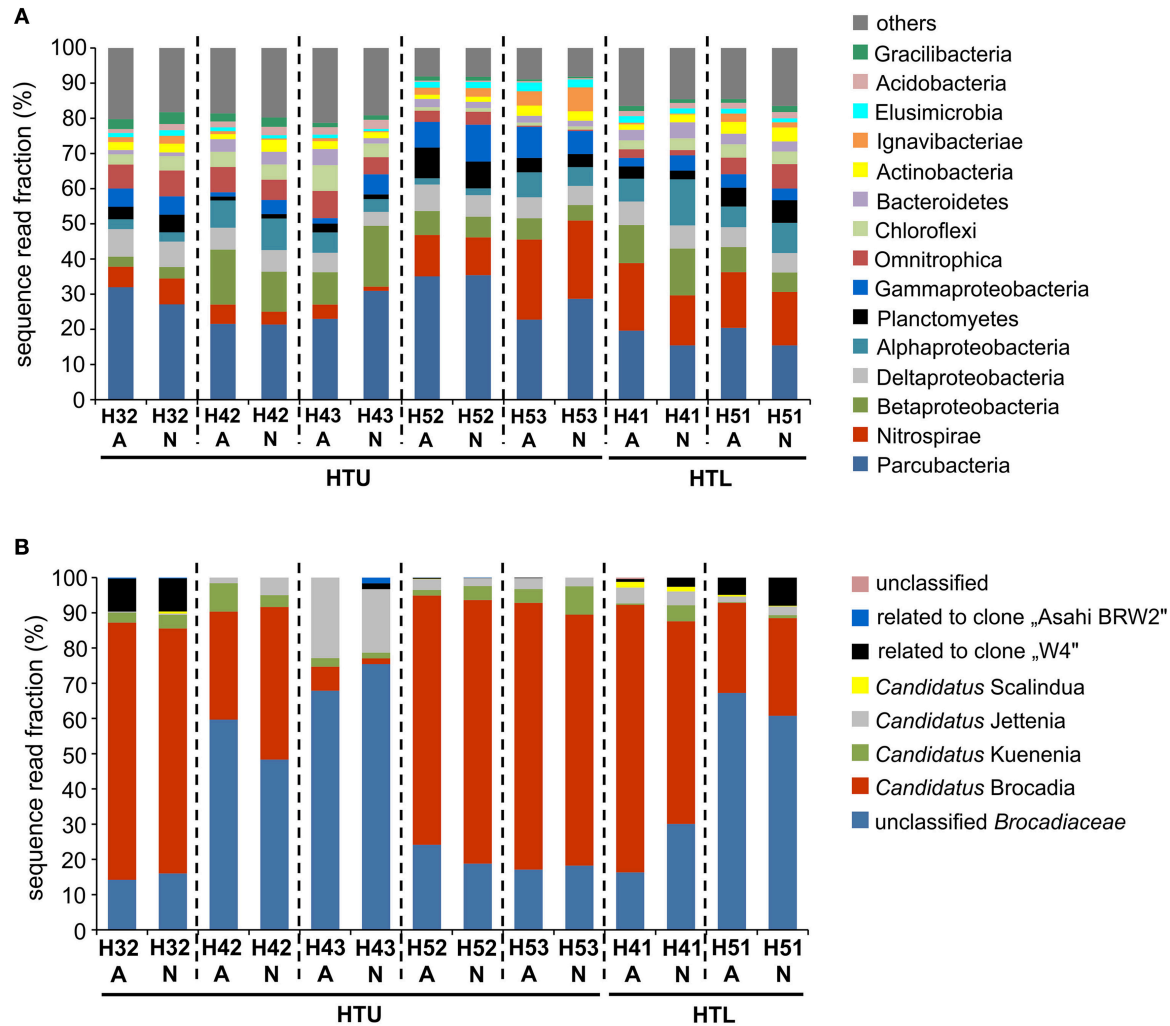
Structure of the total bacterial community **(A)** and of the anammox bacterial community **(B)** based on MiSeq Illumina amplicon sequencing of 16S rRNA genes in the groundwater of seven wells across the two aquifer assemblages, analysis based on metagenomic DNA [August (A) and November (N) 2015]. For **(B)** Bars represent fractions of sequences assigned to different Candidatus genera of anammox bacteria within the *Brocadiaceae* (corresponding to 39 up to 4,264 sequence reads out of 16,383 total bacterial 16S rRNA sequence reads per well).

To complement the functional gene targeted quantitative analysis, groundwater bacterial community structure was assessed based on 16S rRNA gene-targeted MiSeq Illumina amplicon sequencing for samples obtained in August and November 2015. With few exceptions, bacterial communities across both aquifer assemblages were primarily composed of members of Parcubacteria (14–38% of sequence reads) followed by Nitrospirae (1–32%), Betaproteobacteria (3–17%), Deltaproteobacteria (4–12%), and Alphaproteobacteria (1–13%; Figure 4A). Relative fractions of sequence reads affiliated with Planctomycetes ranged from 1 to 9% with highest fractions in the groundwater of anoxic well H52 (4.9–8.8%) followed by 4.6–6.5% in oxic well H51 (Figure 4A). The fraction of sequence reads within the Planctomycetes which were affiliated with anammox-bacteria was lowest in the oxic aquifer (26–33%) and at site H4 of the anoxic upper aquifer assemblage (16–27%) and showed maximum values of 93–96% at wells H53 and H52, indicating that the Planctomycetes community in the groundwater of these wells was almost exclusively composed of anammox bacteria. On the 16S rRNA level representing the bacterial population with protein biosynthesis potential (Blazewicz et al., 2013), the representation of sequence reads affiliated with Planctomycetes was especially high in the groundwater of wells H52 (27%) and H53 (15%), out of which anammox-affiliated reads accounted for 95–96% (Supplementary Figure 5).

16S rRNA gene and transcript-targeted Illumina MiSeq amplicon sequencing identified four candidate genera of anammox bacteria. Among the sequence reads affiliated with *Brocadiaceae*, up to 78% were affiliated with *Candidatus* Brocadia, followed by *Candidatus* Kuenenia and *Candidatus* Jettenia (Figure 4B) with similar results for DNA- and RNA-based sequencing in August 2014 (Supplementary Figure 6A). These results were supported by the *hszA*-targeted cloning approach. Nine different OTUs were observed most of which were affiliated with the *Candidatus* genus Brocadia, with sequence identities of deduced *hzsA* protein sequences with those of *Cand*. Brocadia fulgida ranging from 90 to 92% (Supplementary Figure 6B).

**Figure 5:**
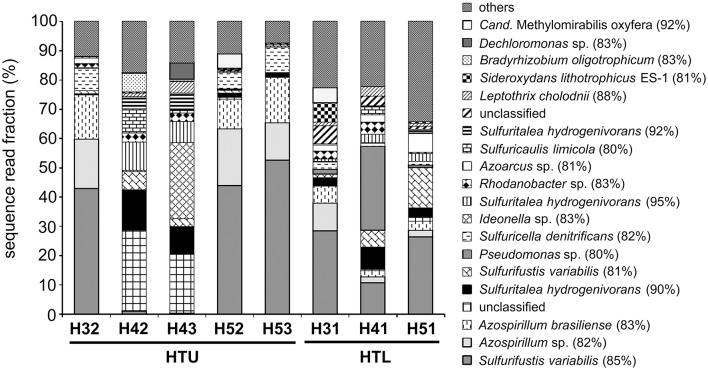
Community structure and taxonomic affiliation of *nirS*-type denitrifiers in the groundwater of the two aquifer assemblages based on Illumina MiSeq amplicon sequencing of *nirS* genes, August 2014. Sequences showing less than 80% *nirS* sequence identity with cultured denitrifiers were referred to as “unclassified.”

MiSeq Illumina amplicon sequencing of the less abundant *nirK* genes resulted in only poor sequence read yields. Consequently, a detailed analysis of denitrifier community composition focused on *nirS*-type denitrifiers only. Except for wells H42 and H43, *nirS*-type denitrifier communities were dominated by one OTU distantly related to *Sulfurifustis variabilis* (nirS-OTU1: 85% sequence identity), and two OTUs related to the genus *Azospirillum* (nirS-OTU2, nirS-OTU3: 82–83% sequence identity). These OTUs accounted for more than 70% of the sequence reads for wells H32, H52, and H53, while they were only represented by few sequence reads at sites H42 and H43 (Figure 5). Here, *nirS*-type denitrifier communities were mainly composed of denitrifiers closely related to *Sulfuritalea hydrogenivorans* (90–95% sequence identity), *Ideonella* sp. (83% sequence identity), and poorly characterized *nirS*-type denitrifiers (nirS-OTU4, sequence identity <80%), with the latter accounting for 20–28% of all the *nirS* sequence reads detected at wells H42 and H43. Since the *nirS* primer set used in this study may discriminate against some denitrifying genera due to mismatches in the primer binding region (Herrmann et al., 2017), we additionally identified potential denitrifiers based on the 16S rRNA sequence information from the same samples (August 2014). This comparison confirmed the presence and distribution patterns of the genera *Sulfurifustis, Sulfuritalea*, and *Sulfuricella* across sites, while it additionally identified the denitrifying genera *Hydrogenophaga* and *Sideroxydans*, which were especially abundant in the groundwater of wells H42 and H43 (*Hydrogenophaga*: 0.09–1.6% of all 16S rRNA gene sequence reads; *Sideroxydans*: 0.1–0.8%).

## Discussion

Reoccurring patterns of ladderane relative concentrations pointed to the existence of potential active sites of anammox in suboxic to anoxic groundwaters of the wells H52 and H53 of the Hainich CZE (Schwab et al., 2017; Figure 2), suggesting a hitherto unrecognized role of anammox in nitrogen cycling in oligotrophic limestone aquifers. Our results not only ultimately proved the occurrence of the anammox process at rates of 3.5–4.7 nmol N_2_ L^−1^ d^−1^ in the groundwater of suboxic to anoxic well H53 but also demonstrated its high relevance compared to denitrification for the removal of fixed nitrogen at an estimated contribution of 83%. Well H53 and the neighboring well H52 showed a very high similarity regarding hydrochemical properties supportive of the anammox process. Moreover, previous findings from PLFA analysis and proteomics provided strong support that active anammox is also ongoing in the groundwater of well H52, where one third of the identified proteins were associated with Brocadiales (Starke et al., 2017). Along with the high representation of anammox-bacteria based on 16S rRNA-targeted Illumina sequencing, maximum abundances of *hzsA* genes, and two orders of magnitude higher *hzsA* compared to *nirS* gene transcripts, these findings provided strong support for these wells being an anammox hotspot within the heterogeneous carbonate-rock aquifer system of the Hainich CZE. Starke et al. (2017) not only demonstrated metaproteomics-based evidence of anammox by *Cand*. Brocadiales at well H52 but found that members of this order were also involved in nitrate reduction to nitrite and to ammonium, nitrogen fixation, ammonification, and CO_2_-fixation. *In situ* nitrite concentrations were usually below the detection limit, suggesting that nitrite was most likely subject to high turnover and could be a limiting factor for *in situ* anammox activity. Hence, we cannot rule out that addition of nitrite in our anammox and denitrification assays might have additionally stimulated anammox but also denitrification activity.

The anammox activity observed in our study is similar to average rates reported from off-shore marine oxygen minimum zones (e.g., 1.9 and 3.0 nmol N_2_ L^−1^ d^−1^; Dalsgaard et al., 2012; Kalvelage et al., 2013) but was substantially lower than anammox activities measured in groundwater contaminated with nitrate or ammonium (Table 1). The strikingly high contribution of anammox to nitrogen loss under oligotrophic conditions agrees with findings from oceanic oxygen minimum zones (Lam and Kuypers, 2011) and with reports from ammonium-contaminated aquifers at low organic carbon availability (<1.0 mg L^−1^ DOC, Smith et al., 2015), while the contribution of anammox was found to be lower (18–41%) in groundwater at DOC concentrations up to 30 mg L^−1^ (Moore et al., 2011; Smith et al., 2015). Consequently, limitation by labile organic carbon was most likely a key factor underlying the observed high relevance of anammox vs. denitrification in the oligotrophic carbonate-rock aquifers of the Hainich CZE. In fact, carbon isotope-based studies in the groundwater of wells H52 and H53 pointed to a tight internal cycling of carbon including oxidation of sedimentary old organic matter depleted in both ^13^C and ^14^C, and subsequent refixation of ^13^C- and ^14^C-depleted CO_2_ by chemolithoautotrophs (Nowak et al., 2017). For the same groundwater sampling wells, Schwab et al. (2017) found a strong depletion of phospholipid [3]- and [5]-ladderanes in ^13^C (δ^13^C values ranging from –48.0 ± 10.5 to –45.9 ± 11.7%0), indicative of active CO_2_-fixation via the acetyl-CoA-pathway and characteristic of anammox bacteria (Schouten et al., 2004), confirming the substantial contribution of the anammox process to *in situ* autotrophic CO_2_-fixation. Disconnection of these groundwater wells from surface-derived organic carbon input further appeared likely due to thick overlying soils, low infiltration potential, and low hydraulic conductivities with estimated groundwater travel times ranging from 295 to 587 years (Kohlhepp et al., 2016; Nowak et al., 2017).

**Table 1:** Anammox and denitrification rates in marine and freshwater environments.

**Study site**	**${\text{NH}}_{4}^{+}$**	**${\text{NO}}_{3}^{-}$**	**Anammox**	**Denitrification**	**References**
	**(μmol L^−1^)**		**(nmol N_2_ L^−1^ d^−1^)**		
Marine anoxic basin	0.2	7.2	24–480 (19–35%)	12–2,568	Dalsgaard et al., 2003
Marine oxygen-deficient water	<0.05	<40	0.4–27 (74–100%)	0.4	Thamdrup et al., 2006
Marine oxygen minimum zone	<0.1	<30	1–21 (35%)	3–190	Dalsgaard et al., 2012
Marine oxygen minimum zone (coastal)	0.25–0.5	<50	2.8–227 (30%)	2.2–5.4	Kalvelage et al., 2013
Freshwater lake	0–53	<0.1–10	24–240 (9–13%)	498–2,322	Schubert et al., 2006
Nitrate-contaminated groundwater	10.6–145	3,000–7,172	N/A	387,000–465,000	Tobias et al., 2001
Fertilizer-contaminated groundwater (DOC up to 30 mg L^−1^)	0.5–19,680	3.2–3,854	~319–751 (18–36%)	N/A	Moore et al., 2011
Wastewater-contaminated groundwater (DOC < 1.0 mg L^−1^)	0–47	0–209.4	9.1–458 (39–90%)	1.0–662	Smith et al., 2015
Carbonate-rock aquifers (DOC < 1.8 mg L^−1^)	3.4–30	12–572	3.5–4.7 (83%)	0.7	This study

*N/A, no data given in reference. Estimated contribution of anammox to total N_2_ production given in parentheses*.

Under *in situ* conditions, nitrite fueling the anammox process could originate from nitrate reduction by anammox bacteria or other nitrate reducers but also from incomplete nitrification. Oxygen concentrations of 0–2.2 μmol L^−1^ in the groundwater of suboxic well H53 may provide conditions supportive of a coupling between aerobic and anaerobic ammonium oxidation as described from marine oxygen minimum zones (Lam et al., 2007, 2009; Lam and Kuypers, 2011) or recently also from intertidal sediments (Fernandes et al., 2016). Detection of transcripts of archaeal and bacterial *amoA* genes in the groundwater of well H53 suggested ongoing aerobic ammonia oxidation at suboxic conditions, albeit at a much lower transcriptional activity compared to oxic well H41, where nitrification rates of 10.4–14.4 nmol NO_x_ L^−1^ d^−1^ were detected in a previous study (Opitz et al., 2014). In line with these results, our molecular analysis identified oxygen availability as an important factor driving the predominance of anammox vs. aerobic ammonia oxidation in the genetic potential or transcriptional activity of the groundwater communities across the two aquifer assemblages with anammox being the most favored over aerobic ammonia oxidation at wells H52/H53 and the least at well H41. Here, future studies assessing nitrification and anammox activity in parallel are needed to get more insight into a potential coupling between aerobic and anaerobic ammonium oxidation and its potential effect on overall nitrogen losses from the groundwater of pristine limestone aquifers.

Beyond the identified anammox hotspot, our results have clearly demonstrated the ubiquitous presence of anammox bacteria along the two limestone aquifer assemblages of the Hainich CZE, albeit at a variation of *hzsA* gene abundances by four orders of magnitude across all groundwater wells. Surprisingly, we found *hzsA* gene abundances in the range of 2.3 × 10^5^–3.7 × 10^6^ L^−1^ and high *hzsA* transcriptional activity also in the oxic groundwater of wells of the lower aquifer assemblage. While anammox in marine waters, dominated by *Cand*. Scalindua, is inhibited by oxygen levels ≤ ~10 μmol L^−1^ (Jensen et al., 2011; Dalsgaard et al., 2014), it remains unclear if groundwater anammox bacteria could actually thrive and carry out anaerobic ammonium oxidation in the presence of considerable concentrations of oxygen (43–420 μmol L^−1^). Association with aerobic heterotrophs might provide microenvironments at reduced oxygen concentrations. Moreover, a recent study reported a contribution of anammox to *in situ* N_2_ production of up to 58% in permeable riverbeds at an oxygen concentration of 134 ± 14 μmol L^−1^ (Lansdown et al., 2016). Among the four candidate genera of anammox bacteria detected in the two aquifer assemblages, only *Cand*. Brocadia, mostly described from terrestrial habitats (Humbert et al., 2010; Hirsch et al., 2011) and the dominant anammox representative in the communities of our study, has previously been reported to cope with elevated oxygen concentrations of up to 63 μmol L^−1^ (Oshiki et al., 2011).

Denitrification activity observed in this study was in the lower range of rates reported from contaminated aquifers at low labile organic carbon availability or from marine oxygen minimum zones (Table 1) and was substantially lower than rates reported from lake water or from contaminated groundwater at higher organic load. In fact, analysis of the groundwater denitrifier community revealed large fractions of potential autotrophic denitrifiers oxidizing reduced sulfur compounds, hydrogen, or reduced iron such as the genera *Sulfurifustis* (Kojima et al., 2015), *Sulfuritalea* (Kojima and Fukui, 2011), *Sulfuricella* (Kojima and Fukui, 2010), or *Sideroxydans* (Emerson and Moyer, 1997), confirming previous observations of a high genetic potential for sulfur-driven autotrophic denitrification in the aquifers of the Hainich CZE (Herrmann et al., 2015, 2017). Consequently, we cannot rule out that addition of the respective electron donors would have stimulated denitrification in our incubation experiments. However, the low *nirS* transcriptional activity in the groundwater of wells H52 and H53 pointed to low *in situ* denitrification activity. In addition to low availability of suitable organic and presumably also inorganic electron donors, a second factor favoring anammox over denitrification was most likely the constantly low groundwater temperature around 10°C, as studies of both natural and engineered systems found a lower temperature optimum for anammox compared to denitrification (Jetten, 2001; Dalsgaard and Thamdrup, 2002; Rysgaard and Glud, 2004; Dosta et al., 2008; Hu et al., 2013; Lotti et al., 2014).

## Conclusions

Our results have demonstrated a strong functional resemblance between oligotrophic groundwater and marine oxygen minimum zones regarding anammox activity, its high contribution to nitrogen loss, and its potential coupling with aerobic ammonia oxidation. We provided first insight into the quantitative relevance of anammox vs. denitrification in pristine groundwater. Together with knowledge gained from previous PLFA- and proteomics-based studies, our results point to the existence of an anammox hot spot within the heterogeneous carbonate-rock aquifer system of the Hainich CZE, where anammox dominates nitrogen cycling in suboxic to anoxic groundwater zones and could also make a substantial contribution to autotrophic CO_2_-fixation under conditions of strong organic carbon limitation. Future studies will address whether coupling between anammox and nitrification enhances nitrogen loss from oligotrophic groundwater environments and will elucidate potential mechanisms which allow anammox bacteria to thrive in oxic groundwater.

## Author contributions

MH, KK, and ST designed the work. SK performed most of the molecular work, contributed to chemical analysis and field work, analyzed all the data, and wrote the first version of the manuscript. ST and KT contributed substantially to the biogeochemical, transect-oriented interpretation of the results. Analysis of sequence data was carried out by SK and MH. PG performed a large part of the RNA-based work. BT carried out the ^15^N based assessment of anammox and denitrification rates. VS performed the analysis of [3]-ladderane and [5]-ladderane phospholipid derived fatty acids. All authors contributed to the writing of the manuscript.

### Conflict of interest statement

The authors declare that the research was conducted in the absence of any commercial or financial relationships that could be construed as a potential conflict of interest.
