# Supplementary material for: Nitrogen Loss from Pristine Carbonate-Rock Aquifers of the Hainich Critical Zone Exploratory (Germany) Is Primarily Driven by Chemolithoautotrophic Anammox Processes
Source: Front Microbiol. 2017 Oct 10;8:1951. doi: 10.3389/fmicb.2017.01951 (PMC5641322; doi:10.3389/fmicb.2017.01951)
Supplement: Supplementary file 1 [file Image1.PDF]

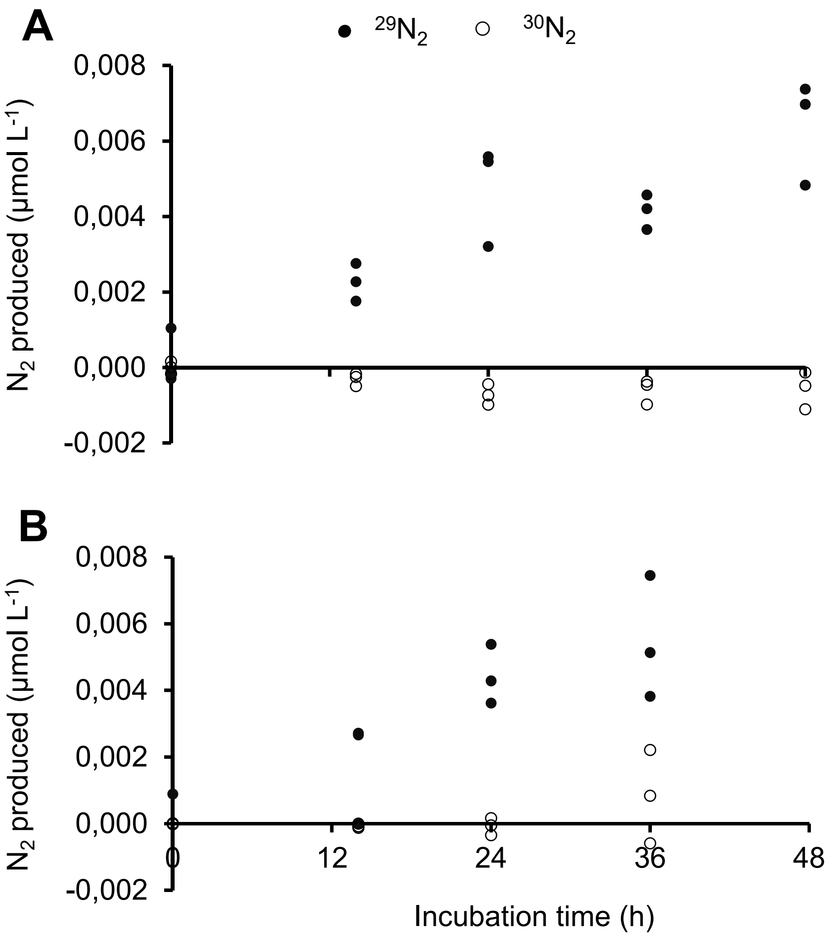

Supplementary Figure 1. Formation of $^{29}N_2$ (filled circles) and $^{30}N_2$ (open circles) over a 48 hours incubation period in anammox and denitrification assays. (A) Incubation with $^{15}N\text{-}NH_4^+$ and $^{14}N\text{-}NO_2^-$. (B) Incubation with $^{15}N\text{-}NO_2^-$ and natural $^{14}N\text{-}NH_4^+$ background. Assays were run in triplicates.
