# Supplementary material for: Nitrogen Loss from Pristine Carbonate-Rock Aquifers of the Hainich Critical Zone Exploratory (Germany) Is Primarily Driven by Chemolithoautotrophic Anammox Processes
Source: Front Microbiol. 2017 Oct 10;8:1951. doi: 10.3389/fmicb.2017.01951 (PMC5641322; doi:10.3389/fmicb.2017.01951)
Supplement: Supplementary file 2 [file Image2.PDF]

A

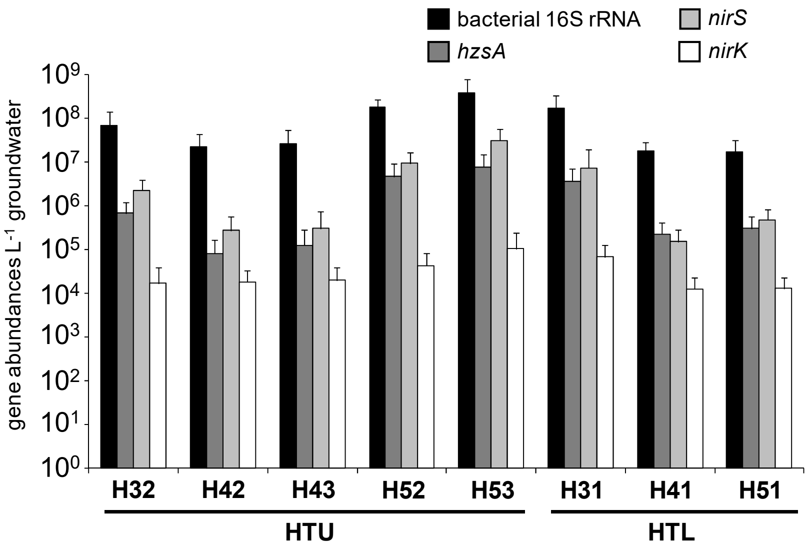

B

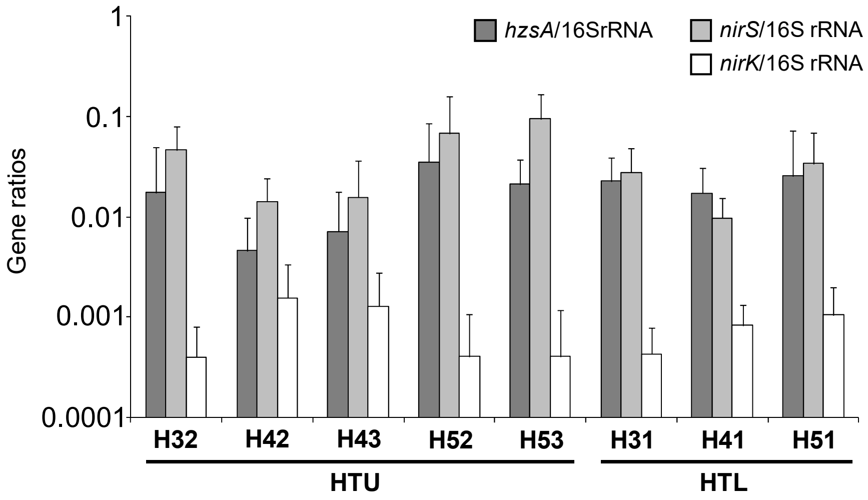

Supplementary Figure 2. Total abundances (A) and gene ratios relative to bacterial 16S rRNA genes (B) of bacterial 16S rRNA genes, *hzsA* , *nirS* and *nirK* genes in groundwater samples obtained from eight wells of the upper and lower aquifer assemblage. Bars represent mean (± standard deviation) of monthly measurements from January 2014 to June 2015.
