# Supplementary material for: Nitrogen Loss from Pristine Carbonate-Rock Aquifers of the Hainich Critical Zone Exploratory (Germany) Is Primarily Driven by Chemolithoautotrophic Anammox Processes
Source: Front Microbiol. 2017 Oct 10;8:1951. doi: 10.3389/fmicb.2017.01951 (PMC5641322; doi:10.3389/fmicb.2017.01951)
Supplement: Supplementary file 3 [file Image3.PDF]

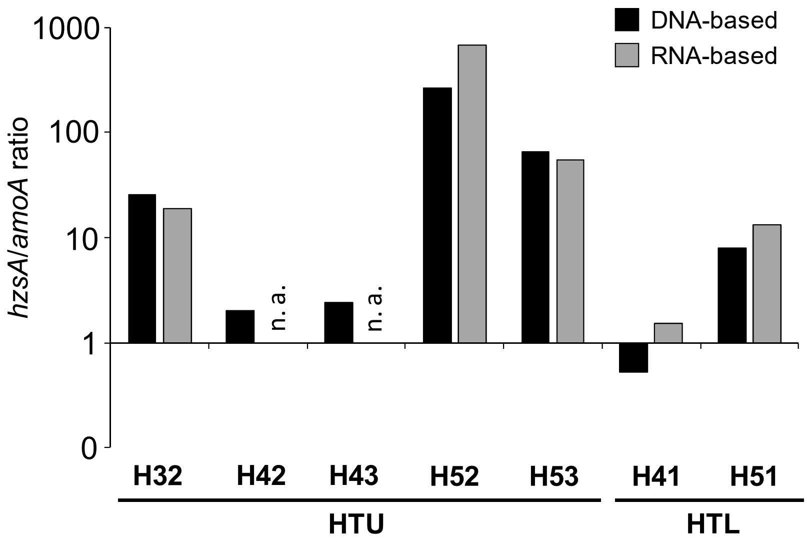

Supplementary Figure 3. Ratios of *hzsA*/archaeal + bacterial *amoA* on the gene and the transcript level in groundwater samples of the two aquifer assemblages. Data are means of two time points (August and November 2015). n. a. = not analyzed
