# Supplementary material for: Nitrogen Loss from Pristine Carbonate-Rock Aquifers of the Hainich Critical Zone Exploratory (Germany) Is Primarily Driven by Chemolithoautotrophic Anammox Processes
Source: Front Microbiol. 2017 Oct 10;8:1951. doi: 10.3389/fmicb.2017.01951 (PMC5641322; doi:10.3389/fmicb.2017.01951)
Supplement: Supplementary file 4 [file Image4.PDF]

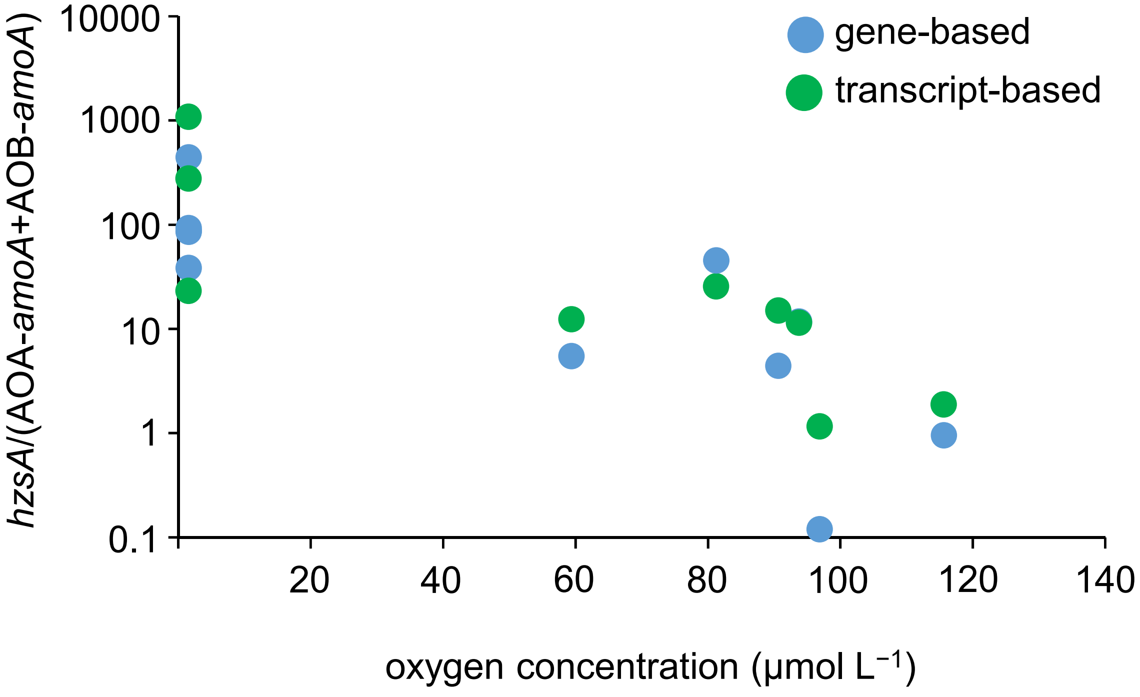

Supplementary Figure 4. Ratios of [*hzsA*/sum of archaeal and bacterial *amoA*] plotted against the oxygen concentration in the respective groundwater sample. Data are based on ten groundwater samples obtained from five groundwater wells in August and November 2015. Blue dots: DNA-based analysis. Green dots: Transcript-based analysis.
