# Supplementary material for: Nitrogen Loss from Pristine Carbonate-Rock Aquifers of the Hainich Critical Zone Exploratory (Germany) Is Primarily Driven by Chemolithoautotrophic Anammox Processes
Source: Front Microbiol. 2017 Oct 10;8:1951. doi: 10.3389/fmicb.2017.01951 (PMC5641322; doi:10.3389/fmicb.2017.01951)
Supplement: Supplementary file 5 [file Image5.PDF]

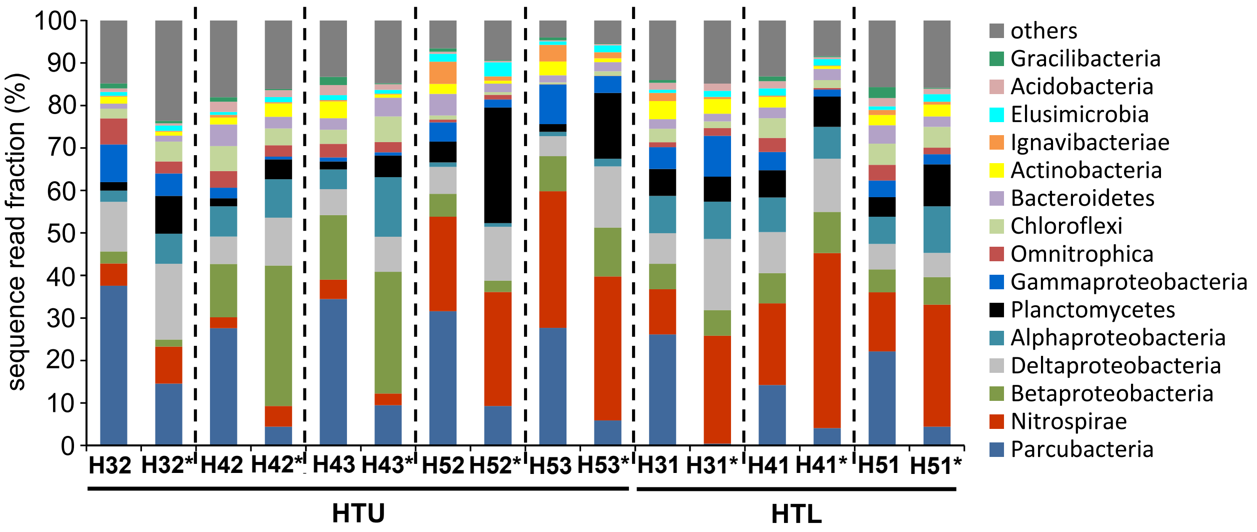


**Supplementary Figure 5.** Bacterial community structure based on MiSeq Illumina amplicon sequencing of 16S rRNA genes in the groundwater of eight wells across the two aquifer assemblages, analysis based on metagenomic DNA and RNA (indicated with *) (August 2014).
