# Supplementary material for: Nitrogen Loss from Pristine Carbonate-Rock Aquifers of the Hainich Critical Zone Exploratory (Germany) Is Primarily Driven by Chemolithoautotrophic Anammox Processes
Source: Front Microbiol. 2017 Oct 10;8:1951. doi: 10.3389/fmicb.2017.01951 (PMC5641322; doi:10.3389/fmicb.2017.01951)
Supplement: Supplementary file 6 [file Image6.PDF]

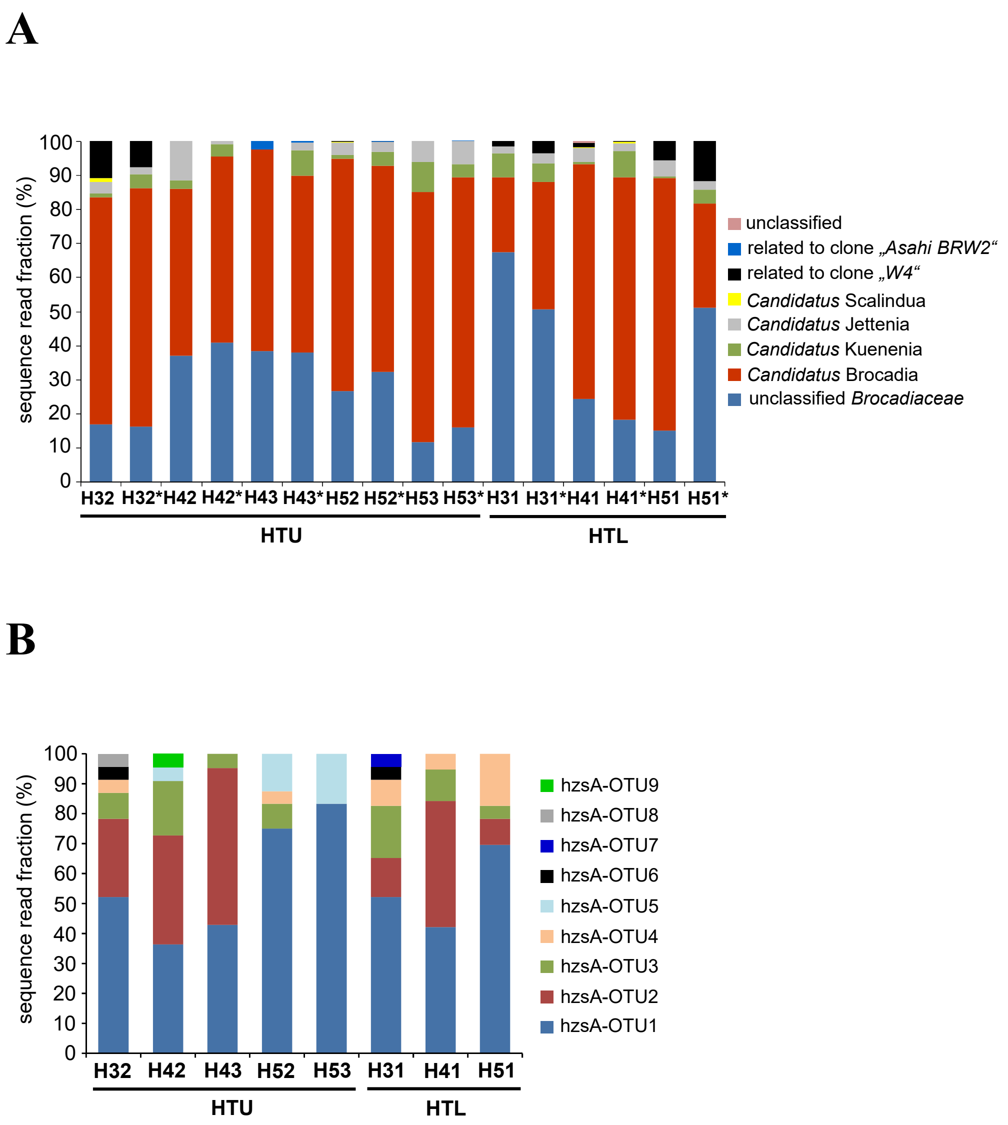

Supplementary Figure 6. Community structure of anammox bacteria based on MiSeq Illumina sequencing of 16S rRNA genes (A) and relative fractions of OTUs of anammox bacteria based on deduced hzsA protein sequences (B) in the groundwater of the two aquifer assemblages. For (A), sequencing was based on metagenomic DNA and RNA (indicated with *). For (B), sequence identities (protein level) to closest cultured relatives are: OTU1, OTU2, OTU3, OTU4: *Cand.* Brocadia fulgida (92%), OTU5: *Cand.* Brocadia fulgida (91%), OTU6: *Cand.* Jettenia asiatica (91%), OTU7: *Cand.* Jettenia asiatica (88%), OTU8: *Cand.* Brocadia fulgida (90%), OTU9: *Cand.* Jettenia asiatica (91%). A total of 169 cloned *hzsA* sequences were analyzed with the number of sequences per groundwater well ranging from 19 to 23.
