# Supplementary material for: Nitrogen Loss from Pristine Carbonate-Rock Aquifers of the Hainich Critical Zone Exploratory (Germany) Is Primarily Driven by Chemolithoautotrophic Anammox Processes
Source: Front Microbiol. 2017 Oct 10;8:1951. doi: 10.3389/fmicb.2017.01951 (PMC5641322; doi:10.3389/fmicb.2017.01951)
Supplement: Supplementary file 7 [file Table1.pdf]

Supplementary Table 1. Physicochemical parameters of groundwater samples from eight wells of the Hainich aquifer assemblages. Data represent mean (± standard deviation) of samples obtained between January 2014 and June 2015 (n = 19).

Well	H32	H42	H43	H52	H53	H31	H41	H51
depth (m)	22.5	12.7	12	65	50	47	48	88
pH	7.3±0.1	7.1±0.1	7.1±0.1	7.3±0.1	7.3±0.1	7.3±0.1	7.3±0.2	7.2±0.1
temperature (°C)	9.9±0.7	9.5±0.2	9.6±0.2	10.4±0.2	10.2±0.5	10.4±0.9	10.2±0.3	10.5±0.3
oxygen (µmol L^{-1})	63±15	0.1±0.2	0.0±0.1	0.0±0.1	0.4±0.7	242±81	170±26	91±8
redox potential (mV)	401.2±47.9	157.5±15.8	241.8±15.9	228.9±39.4	262.8±37.8	402.5±66.3	407.2±49.2	416.2±49.2
DOC (mg L^{-1})	1.8±0.7	1.7±0.8	1.8±0.6	1.6±0.8	1.6±0.6	1.7±0.8	1.5±0.7	1.8±0.7
TOC (mg L^{-1})	2.2±0.9	1.9±0.8	2.0±0.6	1.9±0.9	1.9±0.7	2.1±1.0	1.7±0.7	1.8±0.9
NH_4^+ (µmol L^{-1})	6.0±6.6	11.0±6.9	7.8±5.6	22.2±13.9	30.0±17.3	3.0±3.5	6.0±4.2	3.4±3.8
NO_3^- (µmol L^{-1})	572.3±247.7	13.9± 27.9	11.7±28.1	29.6±29.1	31.3±28.4	425.4±138.2	149.9±47.1	117.9±45.1
NO_2^- (µmol L^{-1})	0.3±0.2	0.3±0.5	0.1±0.2	0.2±0.2	0.2±0.2	0.2±0.2	0.3±0.6	0.3±0.5
SO_4^{2-} (µmol L^{-1})	633.7±177.4	319.1±74.3	325.2±63.4	843.7±255.4	596.4±196.6	1016.6±29.9	1079.7±335.9	2525.1±692.7
K (µmol L^{-1})	71.3±2.4	168.7±4.0	143.9±3.3	253.5±6.8	353.5±26.8	77.2±10.2	116.0±29.2	47.1±4.8
Ca (mmol L^{-1})	2.35±0.06	2.13±0.21	2.21±0.16	1.72±0.03	1.54±0.11	2.42±0.11	2.80±0.23	4.55±0.15
