# Supplementary material for: Nitrogen Loss from Pristine Carbonate-Rock Aquifers of the Hainich Critical Zone Exploratory (Germany) Is Primarily Driven by Chemolithoautotrophic Anammox Processes
Source: Front Microbiol. 2017 Oct 10;8:1951. doi: 10.3389/fmicb.2017.01951 (PMC5641322; doi:10.3389/fmicb.2017.01951)
Supplement: Supplementary file 8 [file DataSheet1.PDF]

Supplementary Material

Nitrogen loss from pristine carbonate-rock aquifers of the Hainich Critical Zone Exploratory (Germany) is primarily driven by chemolithoautotrophic anammox processes

Swatantar Kumar, Martina Herrmann, Bo Thamdrup, Valérie F. Schwab, Patricia Geesink, Susan E. Trumbore, Kai-Uwe Totsche, Kirsten Küsel*

*** Correspondence:** Kirsten Küsel: kirsten.kuesel@uni-jena.de

Supplementary Material contains:

Supplementary Figure 1

Supplementary Figure 2

Supplementary Figure 3

Supplementary Figure 4

Supplementary Figure 5

Supplementary Figure 6

Supplementary Table 1
